# A Scoping Review of Nature, Land, and Environmental Connectedness and Relatedness

**DOI:** 10.3390/ijerph18115897

**Published:** 2021-05-31

**Authors:** Samantha Keaulana, Melissa Kahili-Heede, Lorinda Riley, Mei Linn N. Park, Kuaiwi Laka Makua, Jetney Kahaulahilahi Vegas, Mapuana C. K. Antonio

**Affiliations:** 1 Office of Public Health Studies, Thompson School of Social Work and Public Health, University of Hawai‘i at Mānoa, Honolulu, HI 96822, USA; sherrera@hawaii.edu (S.K.); mkahili@hawaii.edu (M.K.-H.); lorindar@hawaii.edu (L.R.); kuaiwi@hawaii.edu (K.L.M.); jetney@hawaii.edu (J.K.V.); 2 Native Hawaiian and Indigenous Health, Office of Public Health Studies, Thompson School of Social Work and Public Health, University of Hawai‘i at Mānoa, Honolulu, HI 96822, USA; 3 Health Sciences Library, John A. Burns School of Medicine, University of Hawai‘i at Mānoa, Honolulu, HI 96822, USA; 4 Department of Social Work, Thompson School of Social Work and Public Health, University of Hawai‘i at Mānoa, 1960 East-West Road, Honolulu, HI 96822, USA; meip@hawaii.edu

**Keywords:** nature, land, environment, cultural connectedness, spirituality, psychometrics, systematic review

## Abstract

The importance of nature and the environment in relation to human health is coalescing, as demonstrated by the increased research that attempts to measure nature connectedness and relatedness. These findings align with constructs of cultural connectedness that assess for land connectedness as part of Indigenous ways of knowing. From an Indigenous worldview, relationships with the environment are critical to wellbeing. The purpose of this comprehensive systematic scoping literature review was two-fold: (1) identify and summarize existing measures of land, nature, and/or environmental connectedness, relatedness, and attitudes and (2) evaluate the psychometric properties of these scales. In total, 1438 articles were retrieved from select databases including PubMed/MEDLINE, PsycINFO, CINAHL (EBSCO), and Academic Search Complete (EBSCO). The final searches and application of the inclusion/exclusion criteria resulted in 57 unique articles and 38 scales categorized as connectedness and relatedness scales (*n* = 9 scales), attitudinal and values-based scales (*n* = 16 scales), cultural and spiritually based scales (*n* = 9 scales), and paradigm-based scales (*n* = 4 scales) (articles could be placed in multiple categories). Psychometric properties and general outcomes associated with nature-related scales are reported, with implications for future education, research, practice, and policy.

## 1. Introduction

An understanding of the role of nature to human wellbeing is beginning to emerge, as evidenced by the growing attention of scholars to this field. In 1984, Wilson laid down the theoretical underpinnings of this movement by publishing Biophilia, which hypothesized that humans tend to seek connection with nature and other life forms. The term philia can be traced back to Aristotle who discussed the reciprocal nature of friendship [1]. Since then, the biophilia hypothesis has informed numerous researchers in a variety of disciplines, including those who developed scales to measure different aspects of human’s connection to nature [2].

Over the past thirty years, a significant number of studies related to nature connectedness have been published, resulting in 28 unique scales that were included in our review. Once validated, these scales have been used to assess various aspects of the connection of humans with nature. The most common scales include Connectedness to Nature, a scale that assesses nature as a source of happiness [3]; Nature Relatedness, a scale that measures the strength of connection to nature an individual feels [4]; Inclusion of Nature and Self scale, which measures the impact of one’s connectedness to nature on environmental behavior [5]; and New Ecological Paradigm or New Ecological Paradigm-revised, which is a measure of pro-ecological viewpoints [6].

The approach and, therefore, the dimension of each of the scales vary. The Connectedness to Nature Scale, for example, is a single-factored, 13-item scale that considers the affective dimension of connection to nature [3]. Whereas the Nature Relatedness Scale is presently available as a short-form (6-item) [7] and long-form version [4]. The scale measures the affective, cognitive, and psychomotor or experiential domains through one’s internalized identification, externalized worldview, and physical connection to the natural world, respectively. Numerous other scales were developed, some referencing these core scales and others attempting to expand the utility of prior scales beyond the confines of mental health to such things as climate change, environmental behavior, and physical health.

Although the connection between health and the environment is still being explored, studies have found that having adequate exposure to outdoor environments has a protective factor on the mental health of youth and adults, especially related to stress management [8,9]. Pretty, Peacock, Sellens, and Griffin [10] found physical benefits, such as exercising outdoors, so-called “green exercise”, reduced blood pressure and increased mood and self-esteem more than indoor exercise. Other studies have found that engaging in outdoor activities has a beneficial effect on individuals with ADHD [11]. Furthermore, proximity to nature and greenspace improves the prevalence of childhood asthma [12] and even has a beneficial impact on pain management [13].

The interconnectivity of environmental and human health is further supported by recent climate change literature indicating that rising surface temperatures are responsible for the increased number of droughts and stronger intensity of storms [14]. Furthermore, warmer ocean temperatures have created a deadly cycle of environmental degradation, including increased ocean acidification, reduced biodiversity, threatened economic and food security, and reduced human enjoyment [15]. The impacts of climate change detrimentally affect human safety, security, and ability to enjoy nature.

The United Nations Permanent Forum on Indigenous Issues stated that “climate change exacerbates the difficulties already faced by vulnerable populations [16]”. Indigenous communities globally have been especially hard hit by long-standing colonial environmental mismanagement. Indigenous people see themselves and nature as part of an extended kinship network, viewing themselves as related to nature and thus inextricably intertwined with the health of the environment around them [17]. This differing perception of the environment has led Indigenous people to play a significant role in combating climate change [18], expanding knowledge of conservation strategies [19], and recognizing the interconnectivity of land to human wellbeing [20].

Scales specifically relevant to Indigenous peoples’ conception of nature connectedness were identified as an area for growth. One such scale that was included in this study is the Cultural Connectedness Scale, which was developed to understand the role of cultural connectedness as a protective factor for First Nation Indigenous peoples [21]. This multidimensional construct consists of 29 items and is centered around the dimensions of identity, traditions, and spirituality. Other examples of scales that incorporate Indigenous conceptions or ways of knowing include the Aboriginal Cultural Engagement Scale, Awareness of Connectedness Scale, and the Hawaiian Cultural Scale [22,23,24]. These scales recognize the role land plays in culture and wellbeing.

While the literature provides significant discussion of the benefits to humans of engaging in nature and the benefits humans can have on nature through proper care, minimal literature explicitly discusses the reciprocal relationship between nature and humans. In the same vein, Indigenous land constructs are largely absent in the literature. Although general connectedness scales were applied to Indigenous people, there were no specific land-connected scales developed using Indigenous ontologies. Nonetheless, studies where the population was Indigenous, those that incorporated the cultural aspects of land, and that included an element of Indigenous spiritual connection were included. Despite this inclusion, a future vein of nature connectedness studies focusing on Indigenous conception of nature should be explored.

Previous research confirms the importance of nature connectedness (i.e., through the Connectedness to Nature Scale [3]) and nature relatedness (i.e., through the Nature Relatedness Scale [4,7]), with increased connection and relatedness being associated with positive wellbeing [3]. These findings align with other Indigenous constructs of cultural connectedness and resilience and should be further explored as related factors. Measurement of these items are particularly important to ensure constructs have been validated for multiple populations, while capturing relationships that exist between nature, the environment, wellbeing, and multi-dimensional constructs of resilience.

### Purpose

The purpose of this comprehensive scoping literature review was two-fold. First, this review aimed to identify and summarize existing measures of land, nature, and/or environmental connectedness, relatedness, and attitudes using select databases including PubMed/MEDLINE, PsycINFO, CINAHL (EBSCO), and Academic Search Complete (EBSCO). The second purpose of this study was to evaluate the psychometric properties of the identified scales that resulted from the comprehensive search. This resulted in the following research questions: What are the existing measures and constructs of nature or land connectedness and relatedness (including attitudes related to nature connectedness and relatedness)? What are psychometric properties of existing measures, including goodness of fit statistics, reliability, and validity properties? What variables have been associated with nature or land connectedness?

## 2. Materials and Methods

The scoping review team consisted of a core team of subject matter specialists (in public health related to native Hawaiian health, historical trauma, and policy) and a secondary team of public health student data extractors. The research questions were developed by the first and last author, and the inclusion and exclusion criteria were refined as a team.

The scoping review was conducted according to the PRISMA-ScR guidelines [25], but the team did not register a protocol for the review. Based on eligibility criteria, articles included in the review referenced various types of measurements and scales that relate to nature connectedness, including scales that referenced connecting with nature/environment as a result of cultural connectedness (specific to Indigenous communities), scales that assessed for spirituality that included connection with nature, scales that assessed nature and environmental attitudes, and scales that assessed for nature connectedness indirectly through moral expansiveness, spiritual, or cultural connectedness. Excluded articles included papers that did not cite/include a specific scale, scales or articles that were not in English, articles that focused on neighborhood connectedness, and/or articles related to issues of climate change and environmental consumer behaviors, as those are topics beyond the scope of this review.

### 2.1. Data Collection and Search Strategy

The final search for this review was conducted on 20 March 2020. We searched the following databases: PubMed/MEDLINE, PsycINFO, CINAHL, and Academic Search Complete. The PubMed/MEDLINE, PsycINFO, and CINAHL databases selected were chosen based on their relevance to health and well-being, and Academic Search Complete was selected for its broad range of interdisciplinary content. There were no limitations on dates, ages of study participants, or subject area. The search strategy employed the use of various combinations of search terms related to nature and culture connectedness in relation to health, identity, and values, in addition to terms for scale development measurements, assessments, and surveys and questionnaires (refer to Appendix A for a copy of the search strategy employed).

### 2.2. Study Selection

**Figure 1:**
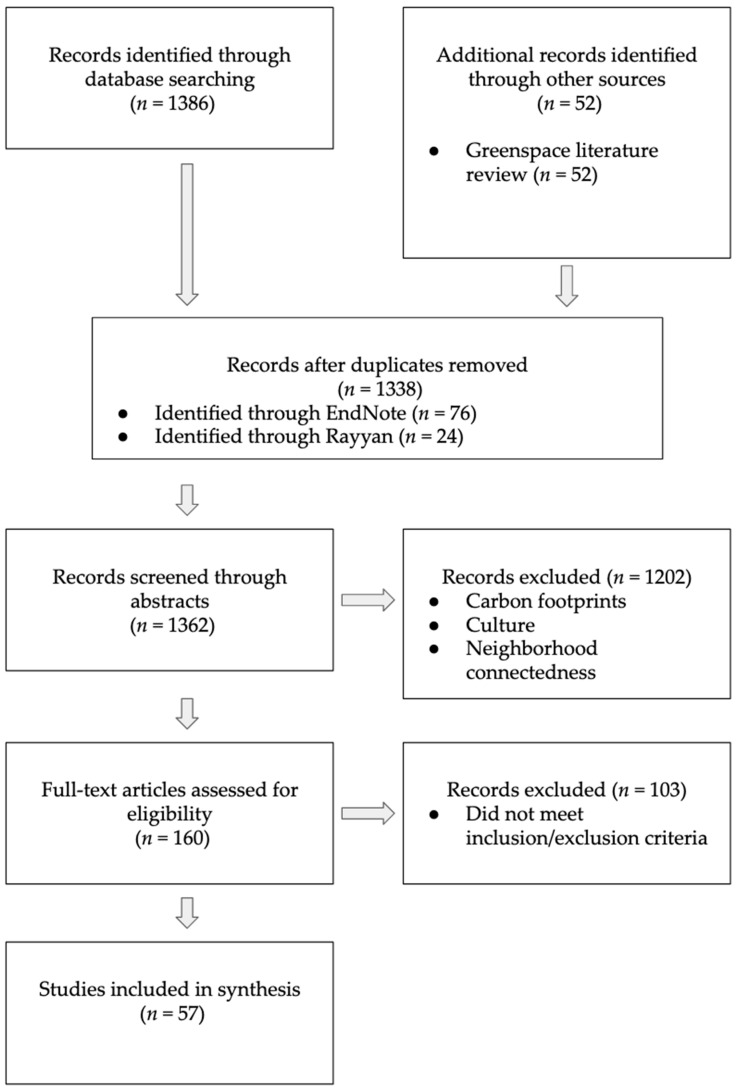
PRISMA Flowchart Diagram.

The final database search yielded 1386 records, and handsearching added another 52 (refer to Figure 1. PRISMA Flowchart Diagram). Titles and abstracts were screened by three team members using the Rayyan QCRI Systematic Review web application [26]. Disagreements were resolved through discussion. Full-text and data abstraction was conducted by four members. Inter-rater reliability was piloted and calculated at 85% between the four team members based on ten articles we reviewed as a group. Full-text review and data abstraction was split between two teams of two members with an inter-rater reliability score of 90% and 83%.

During the full-text review process, the research team extracted key data from articles, with a primary focus on scales that measured land, nature, and environmental connections and relationships. Scales that were the primary focus of the study (i.e., an independent or dependent variable of a study) were included in the data extraction process. The scales and dimensions were reviewed, and authors identified four major categories: (1) connectedness and relatedness, (2) attitudes and values, (3) cultural and spiritually based scales, and (4) environmental paradigm-based scales. Individual items and factors were then assessed for each scale to determine the appropriate category. If items from a scale assessed multiple categories, the scale was classified based on the order of the categories cited. For instance, if a scale measured connectedness to land and attitudes about the environment, the scale was ultimately categorized as a connectedness or relatedness scale.

### 2.3. Psychometric Analyses

Articles were reviewed for their reporting of psychometric properties. First, reliability and validity were assessed based on authors indicating any form of reliability (i.e., internal reliability, test-retest reliability) and/or validity (i.e., content validity, construct validity) for their sample of their study. Reliability that included Cronbach’s alpha or McDonald’s Omega was marked as “acceptable” if values were equal or greater than 0.70. Validity was marked as “acceptable” if the authors reported good content, convergent, or divergent validity.

Next, the research team determined whether factor analyses were conducted for the sample of their study and whether goodness-of-fit statistics were provided. Goodness-of-fit statistics were reported as “acceptable” based on the information listed in the manuscript and only for constructs with the nature or land connectedness items. For instance, in instances when the goodness-of-fit statistics were provided, the team prioritized the value of RMSEA (0.08 or less), followed by CFI/TLI (0.95 or greater), and lastly, other goodness-of-fit statistics including chi-square and SRMR (0.08 or less) [27,28]. If all values were considered within the “acceptable” range, the measure was marked as “acceptable” for goodness-of-fit statistics. On the other hand, if the RMSEA was “unacceptable” despite all other goodness-of-fit statistics meeting criteria, the team marked the construct as “unacceptable” for goodness-of-fit statistics.

The final searches with the inclusion and exclusion criteria applied resulted in 57 final studies, which were included in the analyses of this scoping review. Upon review of the final scales, the research team classified the scales as (1) connectedness and relatedness scales (*n* = 11 scales; 35 articles), (2) attitudinal and values-based scales (*n* = 17 scales; 12 articles), (3) cultural and spiritually based scales (*n* = 9 scales; 10 articles), and paradigm-based scales (*n* = 4 scales; 11 articles). Each major category of scales is described in detail below. Refer to Table 1 for a summary of scales by categorization of scales and the number of articles that included each scale.

## 3. Results

### 3.1. Connectedness and Relatedness Scales

Ten different scales measuring some form of nature connectedness and relatedness were identified from forty different studies: Connectedness to Nature Scale (CNS) [3], Connection to Nature Index [29], FlexiTwins Implicit Connectedness with Nature [30], Nature Relatedness Scale (NR-21) [4], Nature Relatedness short form (NR-6) [7], Recalled Nature Connectedness [31], Nature Connectedness as part of the Health Behaviour in School-aged Children study (HBSC) [9], Inclusion of Nature in Self (INS) [5], Extended Inclusion of Nature in Self (EINS) [32], and the Nature Inclusive Measure [33] (refer to Table 1 and Appendix B for detailed information for each study referenced in this section).

There were three measures widely used: the CNS (16 studies), NR-21 (9 studies), and the INS (8 studies). Connectedness and relatedness scales were tested on a wide range of participants ranging from twenty participants [34] to 20,697 participants [9]. Scales were tested in a wide range of countries: Australia [35,36,37,38,39,40,41,42], Austria [43], Canada [4,7,9,34,36,42,44,45,46], China [47], Finland [48], Germany [49], Greece [50,51], New Zealand [36], Poland [52], Scotland [33,53,54], South Africa [33,54], Sweden [55], Switzerland [32], United Kingdom [31,36,42,56,57,58], and the United States [3,30,36,38,42,59,60,61]. The majority of nature connectedness scales were tested in college student populations [3,4,7,32,33,35,36,38,44,45,46,47,49,55]. Three of the studies included tested scales on child-age populations [9,30,37].

All of the connectedness and relatedness scales had at least one study that reported on reliability and validity and/or goodness-of-fit statistics, with the exception of the Connection to Nature Index [37]. Furthermore, several of the scales had studies that reported on reliability and validity measures but did not include goodness-of-fit statistics, including the Connectedness to Nature Scale [3], FlexiTwins Implicit Connectedness with Nature [30], The Nature Relatedness Scale (NR-21) [4,7,41,45,48,55,57,58,61], the Nature Relatedness Short Form (NR-6) Scale [7,36,42], Nature Connectedness (as part of the Health Behaviour in School-aged Children (HBSC) Spiritual Health scale) [9], Extended Inclusion of Nature in Self [32], and Inclusion of Nature in Self (INS) [7,35,36,44,49,53,55,56].

As mentioned, there were a total of 35 articles reporting on the Connectedness to Nature Scale [3]. Of the 35 studies, 23 assessed for reliability and validity, with all 23 demonstrating good reliability and validity. Although some of the studies reported goodness-of-fit statistics for other scales or for an overall structural equation model (SEM), only two of the studies reported the direct goodness-of-fit statistics for the selected connectedness or relatedness to nature scale [31,33]. Moreover, of these two studies, only one met acceptable criteria based on the goodness-of-fit statistics that were reported [33]. The majority of the studies reported the CNS as a unidimensional score despite authors indicating a strong support for a 3-factor model [45,46]. For the NR-21 scale, 13 of the 16 independent studies reported acceptable reliability and validity. Three of the three studies that reported on the Nature Relatedness Short Form (NR-6) indicated good reliability and validity. Only five of the Inclusion of Nature in Self (INS) studies (out of 11 studies) reported reliability and validity measures.

For connectedness and relatedness scales, the most commonly cited outcomes included well-being [3,7,36,39,43,45] and various eco-friendly behaviors [3,47,50,51]. Despite some articles reporting non-significant findings or no changes in outcomes, a collective review of the articles demonstrated favorable results, with increased connectedness or relatedness to nature and the environment. Increased connections to nature based on the Connectedness to Nature Scale [3], Connection to Nature Index [29], FlexiTwins Implicit Connectedness with Nature [30], Recalled Nature Connectedness [31], and Nature Connectedness (as part of the Health Behaviour in School-aged Children (HBSC) Spiritual Health scale) [9,62] was found to be associated with various outcomes, including increased well-being, eco-friendly behaviors, pro-social behavior, recycling, spirituality, self-esteem, life satisfaction, decreased alcohol intake, egoistic concerns, and altruistic concerns. Similarly, increased relatedness to nature based on studies that explored relationships between Nature Relatedness Scale [4] and 6-item short form Nature Relatedness Scale [7] and study outcomes identified positive associations with self-reported health, well-being, hedonic and eudaimonic happiness, lifetime experience with psychedelics, pro-environmental behavior, conscientiousness and openness, and meaning in life and negative associations with dissociative anesthetics and alcohol intake. Inclusion of nature and nature inclusive measures were found to be related to outcomes including Eastern values, nature relatedness, health, happiness, and nature connectedness.

### 3.2. Attitudinal and Values-Based Scales

Sixteen different scales from twelve unique studies were identified measuring environmental attitudes and values: Attitudinal Commitment to Nature-Based Activities [63], Behavioral Commitment to Nature-Based Activities [64], Biospheric Value [65], Children’s Ecological Behaviors [66], Environmental Attitudes [67], Environmental Attitudes Inventory [68], Environmental Behavior [69], Environmental Citizenship [70], Environmental Motives Scale [71], Local Environmental Concern [72], Love and Care for Nature Scale [35], Natural Environments and Feelings About Nature [73], Perceived Importance of the Environment on Health and Well-being (Ropu Kaitiaki) [74], Place Attachment [75,76,77,78], Preferences for Nature Questionnaire (PNQ) [59], and Population and Environment Scale [79] (refer to Table 1 and Appendix C for detailed information for each study referenced in this section).

Scales measuring environmental attitudes and values were tested on participants ranging from 107 participants [80] to 2168 participants [73]. Scales in this category were also administered in multiple countries: Australia [35], Bangladesh [81], Germany [81], Greece [50,51], New Zealand [74], Norway [73], Russia [72] Scotland [53], Singapore [81], Spain [80], United Arab Emirates [82], and the United States [59,64,83]. The majority of studies using attitudes and values scales were tested on adult populations [35,50,51,59,64,73], two studies tested scales with college students [72,82], and two studies tested with child populations [80,81]. Two studies tested specifically with older adult populations: one study specific to adults born between 1920 and 1940 [74] and the other study on adults 55 and older [83].

Almost all of the articles that measured attitudes or values related to nature or the environment reported acceptable reliability or validity. The perceived importance of the environment on health and well-being (Ropu Kaitiaki) [74] was the only study that did not report on any measure of reliability or validity. Furthermore, 10 of the 12 articles reported goodness-of-fit statistics [35,51,59,72,80,81,82]. Of the 10 studies, all 10 reported acceptable goodness-of-fit statistics, including RMSEA, CFI/TLI, and additional goodness-of-fit statistics (i.e., SRMR), which indicates acceptable model representation of the selected scales.

A total of 21 scales measured attitudes and values related to nature, land, and the environment. Collectively, the 21 scales demonstrated favorable outcomes for health, wellbeing, and factors related to health. Of the 21 scales, 6 had studies that did not report outcomes related to the Attitudinal and Values-Based Scales. For studies that did report outcomes, the various outcomes included activism [72], ascription of responsibility and awareness of consequences [83], emotional affinity toward nature, ecological beliefs [80], attitudes on eco-friendly behaviors [50], egoistic/altruistic/biospheric concerns [51], physical activity behaviors [53,73], and well-being [74]. For instance, Zibenberg et al. [72] found that university students in Moscow who reported higher beliefs in biospheric value and local environmental concern also reported higher levels of environmental activism. In addition, Wiles et al. [74] demonstrated in a study involving 671 Maori people that feelings of connectedness to nature were positively associated with well-being.

### 3.3. Cultural and Spiritual Connectedness Scales

In total, 9 different cultural and spiritual connectedness scales were identified: Aboriginal Cultural Engagement Scale, Awareness of Connectedness Scale, Cultural Connectedness Scale, Hawaiian Cultural Scale, Islamic Environmental Consciousness, Multidimensional Model of Maori Identity and Cultural Engagement (MMM-ICE), Pacific Identity and Wellbeing Scale-Revised, Spiritual Attitude and Involvement List, and the Identification with Aboriginal Culture (refer to Table 1 and Appendix D for detailed information for each study referenced in this section).

Cultural and spiritual connectedness scales were primarily administered to Indigenous communities (*n* = 8 or 80% articles). The number of participants ranged from as few as 5 elders [22] to a sample size as large as 3442 youth [24]. The participants’ ages ranged from 11 [84] to 75 [85] years old. One study did not report the ages of participants, and instead, the authors classified participants as elders [22]. Seven of the studies included adolescents or pre-adolescents in their final sample [21,23,24,84,85,86,87], while three studies only included adults over the age of 18 [22,87,88]. The cultural and spiritual scales took place in seven different regions or countries, including Alaska [23], Hawai‘i [24], New Zealand [85,87], Australia [22], Canada [21,84], Muslim countries [88], and the Netherlands [86].

Eight of the nine cultural or spiritual connectedness scale studies assessed for reliability and validity. Based on the standards set by the research team, eight of the nine scales had reliability measures that met “acceptable” criteria. Although the Multidimensional Model of Māori Identity and Cultural Engagement scales demonstrated evidence of construct validity, the subscale that included items related to land connection (i.e., “I feel a strong spiritual association with the land”) did not meet the reliability Cronbach alpha cutoff of 0.7 or higher. Additionally, of the nine cultural or spiritual connectedness scales, only five (56% of scales) assessed for goodness-of-fit statistics. Of the five scales reporting goodness-of-fit statistics, three [21,23,86] met the RMSEA cutoff of 0.08 or less and CFI cutoff of 0.95 or higher, indicating good model fit for the proposed scale factors.

The proposed Awareness of Connectedness Scale (ACS) [23] demonstrated good model fit for the modified second-order four-factor model only, with the proposed factors labeled as Individual, Family, Community, and Natural Environment, with the second-order factor labeled as Awareness. In the original CCS-Identity Scale, Snowshoe and colleagues (2014) suggested a three-factor model comprised of Factor 1: Positive Sense of Exploration and Commitment to One’s Culture, Factor 2: Utility of Traditional Practices and Language, and Factor 3: Connection to the Spirit World through an Adoption of a First Nations Peoples’ Worldview. Since its development, the CCS-Identity Scale has been integrated in other studies. For instance, in the study by Crooks et al. [84], the 11-item CCS-Identity Scale was included in analyses as a unidimensional construct; however, goodness-of-fit statistics were not evaluated. The final factor model of the Spiritual Attitude and Involvement List (SAIL) included the following subscales: (1) Meaningfulness, (2) Trust, (3) Acceptance, (4) Caring for others, (5) Connectedness with Nature, (6) Transcendent Experiences, and (7) Spiritual Activities. These factors were further classified as Connectedness with Oneself, Connectedness with the Environment, and Connectedness with the Transcendent [86].

Of the 10 studies, only 7 reported outcomes that were directly related to the cultural and spiritual connectedness scales. For all seven studies, increased cultural and spiritual connections, including connection to nature or the land, were associated with favorable outcomes. For instance, increased cultural and spiritual connectedness was found to be positively associated with increased connections with oneself [21,23], with one’s culture and cultural values or beliefs [24,87], and with school [21], the environment, and others [86]. Increased connections to culture and spirituality with a nature, land, or environment component was also associated with increased well-being and general life satisfaction [21,84].

### 3.4. Paradigm-Based Scales

Three unique paradigm scales were found (refer to Table 1 and Appendix E for detailed information for each study referenced in this section). Studies using the New Ecological Paradigm Scale [6] were most common, with sample populations of between 60 participants [3] and 468 participants [6]. This scale was administered in multiple countries, including Australia [35,89], the United States [3,7,59,90], Greece [50,51], and Germany [6]. The second paradigm scale, New Ecological Paradigm for Children, specifically focused on minors by modifying the New Ecological Paradigm [91]. This study included 574 male participants between the ages of 6 and 12 years old in Spain [92]. The New Ecological Consciousness [93] represents the third paradigm scale with a study of 184 participants, a majority of whom were undergraduate students [7].

To some degree, all three paradigm scales reported reliability or validity measures. One study [7] reported on the psychometric properties of both the New Ecological Paradigm and New Ecological Consciousness scales. According to Nisbet and Zelenski, both scales demonstrated good validity and reliability, with a Cronbach’s alpha of 0.75 and 0.83, respectively. The New Ecological Paradigm for Children [92] also assessed for reliability and validity. Goodness-of-fit statistics were not reported in these studies. A total of nine studies reported on the New Ecological Paradigm scale. Of the nine studies, five reported reliability and validity outcomes, all of which were considered to be good or acceptable [3,7,35,51,59].

In total, 11 unique articles were included in the final analysis for paradigm-based scales. Of the 11 articles, 7 reported direct or indirect outcomes related to paradigm-based scales. The New Ecological Paradigm Scale was found to be associated with increased use of natural environments for psychological restoration, eco-friendly behaviors (recycling, transportation choices, daily conservation activities), and motivations to engage with nature [94]. Given the focus on children for the New Ecological Paradigm for Children scale, increased levels of pro-environmental attitudes were found to be related to pro-environmental behavior, such as increased energy conservation at home [92]. The New Environmental Paradigm Scale was associated with increased environmental action and biospheric concerns and decreased egoistic and altruistic concerns [3,51,95]. The last paradigm-based scale, the New Ecological Consciousness scale, did not report any outcomes [7].

### 3.5. Readability

The readability of the scales was analyzed, when access to the scale was possible, using the Flesch Reading Ease (FRE) score and the Flesch-Kincaid Grade Level (FKGL) score (refer to Appendix F). FRE and FKGL were generated using Microsoft Word. FRE scores in the 90–100 range are considered very easy, 80–90 is easy, 70–80 is fairly easy, 60–70 is standard, 50–60 is fairly difficult, 30–50 is difficult, and 0–30 is very difficult [96]. The corresponding FKGL are 5th grade (very easy), 6th grade (easy), 7th grade (fairly easy), 8th–9th grade (standard), 10th–12th grade (fairly difficult), 13th–16th grade (difficult), and ≥college graduate (very difficult) [96].

The most highly cited connectedness/relatedness scales, CNS, INS, NR-21, and NR-6, were analyzed. The CNS had an FRE score of 70 (standard) and FKGL of 7. The INS had an FRE score of 40.1 (difficult) and FKGL of 10.1. The NR-21 had an FRE of 67.7 (standard) and FKGL of 6.6. The NR-6 had an FRE of 67 (standard) and an FKGL of 7.1.

The most highly cited attitude/value scales and paradigm-based scales, Children’s Ecological Behavior, NEP, and the NEP for Children, were also analyzed. The Children’s Ecological Behavior had an FRE score of 59.8 (fairly difficult) and FKGL of 6.2. The NEP had an FRE score of 58.4 (fairly difficult) and FKGL of 8.2. The NEP for Children had an FRE of 75.5 (fairly easy) and FKGL of 5.3.

FRE and FKGL were analyzed for all cultural and spiritual connectedness scales items we could access. Scales analyzed include, Aboriginal Cultural Engagement Scale, Awareness of Connectedness Scale, Cultural Connectedness Scale, and the Hawaiian Cultural Scale. The Aboriginal Cultural Engagement Scale had an FRE of 35.6 (difficult) and FKGL of 11.1. The Awareness of Connectedness Scale had an FRE of 61 (standard) and FKGL of 6.9. The Cultural Connectedness Scale had an FRE of 45.9 (difficult) and FKGL of 10.4. Finally, the Hawaiian Cultural Scale had an FRE of 57.2 (fairly difficult) and FKGL of 7.4. 

## 4. Discussion

The first major purpose of this comprehensive scoping review was to summarize existing scales that assess land, nature, and/or environmental connectedness, relatedness, and attitudes. Four broad categories resulted from our exhaustive search, including (1) nature connectedness and relatedness scales, (2) attitudinal and value-based scales, (3) cultural and spiritual scales with nature or land-based items, and (4) paradigm-based scales. The second major purpose of this scoping review was to identify the psychometric properties of scales commonly used to measure nature, land, and environmental connectedness. Nature connectedness, relatedness, and attitudinal scales have been validated in various populations, including children and adults globally. Final studies included in this review spanned locations such as European countries, the United States, Canada, New Zealand, Australia, South Africa, and the United Arab Emirates.

Cultural-based scales were primarily validated with Indigenous populations in Australia, Canada, New Zealand, Alaska, and Hawai‘i. The majority of these scales were also validated with youth or young adults. Articles that focused on culturally based scales described the importance of interconnectedness, not only with land but also with other dimensions, including connections with family, cultural traditions, and cultural practices, while fostering one’s ability to articulate one’s connectedness to culture [21,22,84,87]. Studies that reported on culturally based scales have implications for cultural connectedness at large, including land connectedness as a mechanism for addressing the significant health disparities that continue to persist today amongst Indigenous peoples. In particular, a connection with land, and thus a connection with culture and cultural connectedness, plays an important role in mitigating the negative effects of social and cultural determinants of health experienced by Indigenous peoples. This is not surprising given the growing research that supports the importance of land as a reflection of health amongst Indigenous peoples and thus a relationship with land serving as an indicator of resilience [97,98,99,100,101] despite the large amount of trauma inflicted upon Indigenous peoples due to the ill effects of land displacement. In other cases, these scales were developed with an effort to better understand the general psychometric properties of these scales as well as with the intention of exploring the relationship between cultural connectedness and health in future research.

Spiritually based scales, on the other hand, were mostly validated with young adults from Muslim counties and the Netherlands. Although relationships with nature were described as a different phenomenon from culturally based scales, spiritually based scales identified connectedness with nature as a dimension of facilitating spirituality, while serving as one of the most important coping mechanisms for stressful events [86]. Paradigm-based scales were predominantly validated in the United States, Spain, Germany, Greece, and Australia. Interestingly, paradigm-based scales stemmed from attitudinal scales or nature connectedness scales, such as Connectedness to Nature [3] and Inclusion of Nature in Self [32], or from attitudinal based scales, but they focused on connections to nature from an ecological worldview [51,94]. As such, paradigm-based scales attempt to view connections and attitudes about nature and the environment from a broader perspective, and they address gaps in the literature by taking a systems approach to connecting with nature and the environment.

Overall, the findings from this study demonstrate the importance of connecting with nature or land as a mechanism for improving general health, attitudes, and behaviors. Despite the favorable outcomes for the measures as a whole, findings from this study demonstrate the different conceptualizations of connecting and relating to land. For instance, in the Nature Connectedness Scale [3], the most commonly cited measure of this study, a strong emphasis is placed on an individual’s ability to emotionally connect with the natural world. In the 21-item [4] and 6-item Nature Relatedness Scales [7], the second most common scale cited in this study, items assessed for an individual’s perspective and experience of connecting with nature.

Cultural connectedness scales were generally developed and implemented with Indigenous communities who have intergenerational knowledge, values, and ways of knowing that honor a deep relationship with nature and land. Therefore, cultural connectedness scales intended to capture one’s connection to culture and cultural practices, and they commonly assessed for one’s connection and relationship to/with land. These measures demonstrate the difference in conceptualizing nature-based connections and land-based familial relationships with land. Similarly, spiritually based scales tended to emphasize a holistic connection with land and included items that assessed a connection with others and spirituality as a whole. For instance, the two scales that focused on nature connectedness through spirituality comprised items that assessed one’s enhancement of spirituality through a connection or relationship with nature or land.

Despite that the original intent of this paper was to identify measures that focused on one’s connection, relationship, or attitudes toward nature, land, and/or the environment, the extensive research in spirituality and cultural connectedness, specifically for Indigenous communities, led the research team to expand on these search terms. The expansion of search terms and changes to the inclusion/exclusion criteria allowed for the inclusion of articles that focused on nature, land, and/or the environmental connections through cultural practices and ways of knowing. The expanded search strategy also took an Indigenous lens and approach to land connections, which acknowledges connections to nature and the environment through practices and cultural ways of knowing, such as viewing land as one’s ancestor [97] or viewing food practices as a mechanism that organically facilitates a connection with one’s land.

### Limitations and Future Directions

Despite the strengths of this paper, including a comprehensive review of the literature, there are limitations that must be acknowledged. Similar to other systematic scoping literature reviews, the findings of this study are limited to the inclusion/exclusion criteria set by the research team. One of the criteria excluded papers and scales that were not written in English. Despite this exclusion criterion, the research team identified scales that have been adapted for other non-English-speaking populations. For instance, the CNS [3] has been translated to French and Spanish, which demonstrates the global versatility and usability of these scales. Furthermore, despite the research team taking an approach that aimed to minimize bias during the research process, the lack of specific details about each independent study may have limited the interpretations that were made for each scale and study. Consequently, the findings of study may be limited to the interpretations of the research team and the information directly presented in the selected peer-reviewed journal articles.

Other factors that were not considered in this scoping review include connections to nature or the environment during declared natural disasters, papers that focused exclusively on climate change, or behaviors that focused on the reduction of carbon footprint. Research related to the previously mentioned factors are on the rise due to increasing concerns related to climate change, which have significant considerations for our present connections to land and the environment, as well as due to the implications environmental disasters and climate change may have on future generations. Similarly, this study did not explore connections or relationships with one’s neighborhood or greenspaces, as they were outside the scope of this paper. Therefore, such articles were ultimately excluded from this study. To address this limitation, future scoping or systematic literature reviews should consider expanding on the searches of this study, with consideration given to the factors listed above. Incorporating these variables and search strategies in future research may provide a better understanding of the constructs that exist in relation to these factors and how they may be associated with general outcomes as well as with land, nature, and/or environmental connectedness, relatedness, and attitudes.

## 5. Conclusions

Land, nature, and environmental connectedness are topics that need to be addressed and further explored in relation to improving the health and wellbeing of communities at large. This study demonstrated the diverse measures of nature and land connectedness, with the findings emphasizing the importance of maintaining relationships with nature and land. The findings from this study have several implications. On all levels, including an educational, research, clinical, and policy level, increased connections with nature, land, and the environment at large may enhance one’s overall sense of self and wellbeing. On an educational level, the findings from this study demonstrate the importance of place-based connections as a way to facilitate education at large. Based on these implications, the findings from this study also support the importance of place-based education in school settings [102,103] as well as the importance of land acknowledgement, particularly among Indigenous-serving and land-grant colleges [104]. Schools and educational institutions may particularly play an important role in promoting connections with nature, land, and the environment, while also promoting connections to one’s ancestral lands. These connections may help to address other determinants at large, including institutional racism, by enhancing the connections that one has to land, while fostering a deep sense of responsibility to learn about the land one occupies and creating a deep love for the land [105,106].

On a research and practice level, the findings from this study continue to emphasize the importance of relationships in health, particularly relationships one has with nature, environment, and the land. In terms of the final scales that were identified, the health literacy of each scale demonstrates the importance of readability, with consideration given to the ability of participants to understand the items and questions included in constructs measuring nature and land connectivity. Calculated FRE and FKGL scores for the cultural and spiritual connectedness scales fell into the fairly difficult and difficult ranges more often than other scales. Cultural connectedness scales were more likely to use a combination of English and native languages. FRE and FKGL, as English language constructs, may not be able to adequately assess the readability of those scales. These findings emphasize the importance of developing and implementing constructs for diverse populations.

On a research, practice, and policy level, the findings from this study continue to emphasize the importance of maintaining ties or connections to one’s land. This especially has implications for Indigenous communities, who have experienced significant disconnections to land as a result of colonization, cultural trauma, and historical trauma. Incorporating Indigenous conceptions of nature may expand the scientific understanding of the phenomenon of nature connectedness. Moreover, because Indigenous lifeways are intertwined with nature, the degradation of the environment is particularly harmful and represents a recurring injury to Indigenous people. Thus, Indigenous-focused scales may advance our understanding of how to heal the historical trauma that Indigenous people have experienced. Land reclamation programs, periodic land rest, controlled burns, and other policies that aim to heal the land represent an implicit shift that begins to address the systemic colonial policies that have perpetrated harm on Indigenous lifeways. In turn, these scales may identify land-based cultural practices, which prior studies have shown to be protective factors that simultaneously restore Indigenous wellbeing and promote resiliency. As such, having a better understanding of the ways in which people may foster stronger relationships to the land will help to inform policies that aim to heal trauma through (re)connections with land.

In particular, such scales will quantify the health needs to support various policies, programs, and movements that promote health equity for Indigenous people who have been systematically oppressed through colonialism and Western imperialism. Health equity for Indigenous people requires the understanding of land as a social determinant of health, where the wellness of land is the wellness of people [97,100]. Therefore, the quantification of the intimate relationships with land allows for rigorous, concrete, and Indigenous-centered data to communicate with decision makers the need for reclamation of Indigenous land stewardship. For example, nature connectedness scales might be utilized to support NDN Collective’s Land Back movement that aims to restore ecological health and Indigenous ownership of lands [107]. In addition, nature connectedness scales can support decision-making processes in determining land use and management, especially with regard to sacred spaces such as Mauna Kea and the decision to desecrate it with the Thirty Meter Telescope. Documents, such as cultural, environmental, and health impact assessments, that are utilized for proposed or future projects could use nature connectedness scales to measure the impact of these proposed projects and to re-center the conversation and decision-making on Indigenous health and well-being. Providing data to accurately measure Indigenous health promotes a culture of health in decision-making, where health is a shared value that can foster healing for Indigenous people [108].

## Figures and Tables

**Table 1:** Summary of scales and number of articles for each category of scales *.

Connectedness and Relatedness Scales	Attitudinal and Values-Based Scales	Culturally and Spiritually Based Scales	Paradigm-Based Scales
Connectedness to Nature Scale; (*n* = 15) Connection to Nature Index; (*n* = 1) FlexiTwins Implicit Connectedness with Nature; (*n* = 1) Nature Relatedness Scale (NR-21); (*n* = 9) and Nature Relatedness Short Form (NR-6); (*n* = 3) Recalled Nature Connectedness (single item); (*n* = 1) Nature Connectedness; (*n* = 1) Extended Inclusion of Nature in Self; (*n* = 1) Inclusion of Nature in Self (INS); (*n* = 8) Nature Inclusive Measure; (*n* = 1)	Attitudinal Commitment to Nature-based Activities; (*n* = 1) Behavioral Commitment to Nature-based Activities; (*n* = 1) Environmental Citizenship; (*n* = 1) Biospheric Value (environmental); (*n* = 1) Local Environmental Concern (pollution, hazards); (*n* = 1) Environmental Attitudes; (*n* = 1) The Population and Environment Scale; (*n* = 1) Children’s Ecological Behaviors Scale; (*n* = 1) Environmental Attitudes Inventory; (*n* = 1) Love and Care for Nature Scale; (*n* = 1) Environmental Behavior (*n* = 1) Environmental Motives Scale (eogoistic, altruistic, and biospheric concerns); (*n* = 1) Natural Environments and Feelings about Nature; (*n* = 1) Perceived Importance of the Environment on Health and Well-being; (*n* = 1) Place Attachment; (*n* = 1) Preferences for Nature Questionnaire; (*n* = 1)	Aboriginal Cultural Engagement Scale; (*n* = 1) Awareness of Connectedness Scale; (*n* = 1) Cultural Connectedness Scale; (*n* = 2) Hawaiian Cultural Scale; (*n* = 1) Islamic Environmental Consciousness; (*n* = 1) Multidimensional Model of Maori Identity and Cultural Engagement (MMM-ICE); (*n* = 1) Pacific Identity and Wellbeing Scale- Revised; (*n* = 1) Spiritual Attitude and Involvement List; (*n* = 1) Identification with Aboriginal Culture; (*n* = 1)	New Ecological Consciousness; (*n* = 1) New Ecological Paradigm for Children; (*n* = 1) Revised New Ecological Paradigm (NEP) Scale; (*n* = 7) New Environmental Paradigm Scale; (*n* = 3)

* In the table above, *n* refers to the number of articles that included the referenced scale.

## Data Availability

Data for the articles included in this systematic review are retrievable through PubMed/MEDLINE (https://pubmed.ncbi.nlm.nih.gov/), PsycINFO (https://www.apa.org/pubs/databases/psycinfo), and CINAHL (EBSCO) (https://www.ebsco.com/).

## Appendix A

The final search for this review was conducted on 20 March 2020. We searched the following databases: PubMed/MEDLINE, PsycINFO, CINAHL, and Academic Search Complete. The PubMed/MEDLINE, PsycINFO, and CINAHL databases selected were chosen based on their relevance to health and well-being, and Academic Search Complete was selected for its broad range of interdisciplinary content. There were no limitations on dates, ages of study participants, or subject area. The search strategy employed the use of various combinations of search terms related to nature and culture connectedness in relation to health, identity, and values, in addition to terms for scale development measurements, assessments, and surveys and questionnaires.

PubMed/MEDLINE

(((((((((((“Awareness”[Mesh]) AND nature)) OR ((“Social Environment”[Mesh]) AND nature)) OR ((“Social Identification”[Mesh]) AND nature)) OR “nature relatedness”) OR “sustainable behavior”) OR “environmental attitudes”) OR connectedness) OR (nature AND “Culture”[MAJR]))) AND ((((“Factor Analysis, Statistical”[Mesh]) OR “Reproducibility of Results”[Mesh]) OR ((“Surveys and Questionnaires”[Mesh]))) OR “scale development”)

CINAHL

(“SOCIAL belonging” OR “social identification” OR “ethnic identity” OR “ethnic value” OR “culturally based” OR indigenous) AND (((MM “natural environment”) OR “nature relatedness” OR “nature study” OR connectedness)) AND (“well-being” OR health OR “MENTAL health” OR “MENTAL health services”) AND (survey or questionnaire or instrument or measure or assessment or scale or “test validity”)

PsycINFO

{Ethnic Identity} OR {Ethnic Values} OR {Environmental Attitudes} OR {Culture (Anthropological)} OR {Conservation (Ecological Behavior)}

AND

{Nature (Environment)}

AND

{Psychometrics} OR {Test Construction} OR {Test Reliability} OR {Test Validity}-being

OR

{Ethnic Identity} OR {Ethnic Values} OR {Environmental Attitudes} OR {Culture (Anthropological)} OR {Conservation (Ecological Behavior)}

AND

{Psychometrics} OR {Test Construction} OR {Test Reliability} OR {Test Validity}

AND

health OR well-being

Academic Search Complete

(“SOCIAL belonging” OR “social identification” OR “ethnic identity” OR “ethnic value” OR “culturally based” OR indigenous) AND (((DE “nature”) OR “nature relatedness” OR “nature study” OR connectedness)) AND (“well-being” OR health OR “MENTAL health” OR “MENTAL health services”) AND (survey or questionnaire or instrument or measure or assessment or scale or “test validity” OR “factor analysis”)

## Appendix B

**Table A1:** ***** Summary of articles reviewing connectedness or relatedness to nature.

Last Name of Author(s) Year	General Demographics (*n*, Age, Gender, Location)	Reliability or Validity Reported	Acceptable Reliability or Validity	GOF Statistics Reported	Acceptable GOF Statistics	General Outcomes (↑, ↓, or =)
Connectedness to Nature Scale; Mayer and Frantz 2004 (*n* = 16 articles)						
Barton et al., 2016	*n* = 130, 11 to 18 years, 57 males (43%), 75 female (57%), South Africa and Scotland	N	N/A	N	N/A	↑ female self-esteem
Cervinka, et al., 2012	*n* = 547, 15–87 years, male *n* = 263, female *n* = 284, Austria	N	N/A	N	N/A	↑ well-being
Geng et al., 2015	*n* = 113 university students, 63 male, 50 female, 23–30 years, China	Y	Y	N	N/A	↑ deliberate environmental behaviors = bag usage = college students’ environmental behaviors
Gkargkavouzi 2019	**Study 1 of 2:** *n* = 150, Greek citizens, 87 females, mean age 40.32 (SD = 9.23), Greece	Y	Y	N	N/A	↓ egoistic concerns ↓ altruistic concerns ↑ biospheric concerns ↑ ecological worldview (NEP) ↑ self-construal nature connectedness (INS)
	**Study 2 of 2:** *n* = 400, Greek citizens, 38.6 years (SD = 14.29), 52% female, Greece	Y	Y	N	N/A	↑ support for environmental policies ↑ recycling ↑ consumerism
Howell et al., 2011	**Study 1 of 2:** *n* = 452, average age = 22.17 years (SD = 6.14), 69.4% female, 81.8% Canadian, urban Canadian university	Y	Y	N	N/A	↑ psychological well-being ↑ social well-being = emotional well-being = mindfulness
	**Study 2 of 2:** *n* = 275, average age = 20.39 years (SD = 3.80), 68% female, 89% Canadian, urban Canadian university	Y	Y	N	N/A	↑ psychological well-being ↑ social well-being ↑ emotional well-being ↑ mindfulness-awareness = mindfulness-acceptance
Howell, Passmore, and Buro 2013	**Study 1 of 2:** *n* = 311, ages 18–53, average age = 22.07 years (SD = 6.05), intro psych course students, 68% female, 82% Canadian	Y	Y	N	N/A	↑ well-being ↑ meaning in life
	**Study 2 of 2:** *n* = 227, intro psych course students, ages 18–60, average age 23.29 (SD = 7.67), 63% female, 73% Canadian, Canada	Y	Y	N	N/A	↑ well-being ↑ meaning in life
Kamitsis, Francis 2013	*n* = 190, students, 18–69 years, average age 36.8 (SD = 13.1), 132 females, 58 males, Melbourne, Australia	N	N/A	N	N/A	↑ psychological well-being ↑ spirituality
Lipowski 2019	*n* = 127, karate practitioners with at least 3 years of experience, 18–65 years, average age 21.66 (SD = 11.59), 42 female, 85 male, Poland	N	N/A	N	N/A	↑ sense of comprehensibility ↑ sense of manageability ↑ sense of meaningfulness
Luck et al., 2011	*n* = 1043, southeastern Australia	N	N/A	N	N/A	= neighborhood well-being = personal well-being
Mayer and Frantz 2004	**Study 1 of 5:** *n* = 60, 30 were students, 18 to 68 years, average 31 years (SD = 13), 31 male, 29 female, U.S.	Y	Y	N	N/A	↑ time spent in natural settings
	**Study 2 of 5:** *n* = 102, 42 males, 60 females, introductory psychology students, U.S.	Y	Y	N	N/A	↑ ecological behavior
	**Study 3 of 5:** *n* = 270, students in intro environmental (*n* = 78), psychology (*n*-121), math (*n* = 44), and chemistry (*n* = 27) courses, U.S.	Y	Y	N	N/A	N/A
	**Study 4 of 5:** *n* = 135, 14 to 89 years, mean age 36 years (SD = 19), 31 men, 89 women, and 15 undisclosed gender, predominantly Caucasian (89%), U.S.	Y	Y	N	N/A	↑ environmentalism ↓ consumerism ↑ perspective taking ↑ subjective well-being/life satisfaction
	**Study 5 of 5:** *n* = 57, psychology undergraduates, U.S.	Y	Y	N	N/A	↑ biospheric ↑ ecological behavior ↓ altruistic ↓ egoistic values
McMahan, E.A. and P. Josh 2017	**Study 2 of 2:** *n* = 168 adults, M age = 34.95 (SD = 11.43), 82 female, Amazon’s Mechanical Turk workers, predominantly Caucasian (79%), U.S.	Y	Y	N	N/A	N/A
Moreton et al., 2019	**Study 1 of 2:***n* = 96 67 females Mage = 19.25 years old (SD = 2) first-year psychology university students, Australia	N	N/A	N	N/A	↑ moral elevation ↑ intentions to engage in pro-environmental behavior ↑ willingness to sacrifice for nature
	**Study 2 of 2:***n* = 232, median age 35.66 years old (SD = 12.53), 120 males, U.S., U.K., Australia, Canada, New Zealand	N	N/A	N	N/A	↑ moral elevation ↑ self-transcendent positive emotions ↑ intentions to engage in pro-environmental behavior ↑ willingness to sacrifice for nature
Perkins, HE 2010	**Study 4 of 4:** *n* = 261 tourists, 18–75 years old (average age 41 years), 42% males, 58% females, Australia	Y	Y	N	N/A	↑ pro-environmental behaviors ↑ willingness to make personal sacrifices in order to protect the environment
Whitten et al., 2018	*n* = 26,848 children, average age 11.92 years of age (SD = 0.38), 49.7% females, New South Wales, Australia	N	N/A	N	N/A	↑ self-satisfaction/hedonic well-being ↑ prosocial behavior
Zhang et al., 2014	**Study 1 of 2:** *n* = 1108, 18–88 years, average age 41.08 (SD = 16.56), 44.4% females, 74.8% Caucasian, U.S.	N	N/A	N	N/A	↑ life satisfaction when attuned to nature’s beauty
	**Study 2 of 2:** *n* = 151, 18–78 years, average age 21.39 (SD = 6.94) 73.1% females, 37.7% Caucasian, university students, U.S.	N	N/A	N	N/A	↑ self-esteem when attuned to nature’s beauty
Connection to Nature Index; Cheng and Monroe 2012 (*n* = 1 article)						
Whitten et al., 2018	*n* = 26,848 children, average age 11.92 years of age, 13,364 females, New South Wales, Australia	N	N/A	N	N/A	↑ self-satisfaction ↑ prosocial behavior
FlexiTwins Implict Connectedness with Nature; Bruni et al., 2018 (*n* = 1 article)						
Bruni et al., 2018	*n* = 238 youth, 6 to 15 years, average age 10.33 (SD = 2.14), 132 females, 105 males, Los Angeles (*n* = 170) and Riverside (*n* = 68), U.S.	N	N/A	N	N/A	N/A
Nature Relatedness Scale (NR-21); Nisbet, E.K.; Zelenski, J.M.; Murphy, S.A. 2009 (*n* = 9 articles)						
Beery 2013	*n* = 120, law students, Sweden	N	N/A	N	N/A	N/A
Dean et al., 2018	*n* = 1538, ages 18–70, Brisbane, Australia	N	N/A	N	N/A	↑ NR score and NR experiences report better self-reported health
Forstmann, Sagioglou 2017	*n* = 1487, 18–78 years, mean age 35.77 (SD = 11.88), 913 female, 566 male, 8 other, U.S.	Y	Y	N	N/A	↑ lifetime experience with classic psychedelics ↓ dissociative anesthetics ↑ popular legal drugs ↑ conscientiousness and openness ↑ political conservatism ↑ pro-environmental behavior
Howell, Passmore, and Buro 2013	**Study 1 of 2:** *n* = 311; F = 68%; 82% Canadian; students in an intro psych course at a Canadian university; average age = 22.07 (SD = 6.05), ages 18–53	Y	Y	N	N/A	↑ well-being ↑ meaning in life
	**Study 2 of 2:** *n* = 227; *F* = 63%; 73% Canadian; students in an intro psych course at a Canadian university; average age 23.29 (SD = 7.67) ages 18–60	Y	Y	N	N/A	↑ well-being ↑ meaning in life
Lumber, Richardson, Sheffield 2017	**Study 1 of 3:** *n* = 203, 18–66 years, mean age 36.90 years (SD = 13.16), 145 female, 175 U.K. residents	Y	Y	N	N/A	↑ engagement and valuing humanistic and moralistic indicators
	**Study 2 of 3:** *n* = 118, 18–78 years, mean age 38.76 years (SD = 15.32), 79 female, 104 U.K. residents	Y	Y	N	N/A	↑ engagement and valuing humanistic and moralistic indicators
	**Study 3 of 3:** *n* = 72 participants (14 male) with a mean age of 23.93, ranging from 18 to 57 years old	Y	Y	N	N/A	↑ engagement with nature’s aesthetics ↑ moralistic (compassion) value
Lyons, Carhart-Harris 2018	*n* = 14, moderate to severe MMD, 64.3% men, average age 45.8, 78.6% Caucasian, U.K.	N	N/A	N	N/A	↑ psychologically supportive administration of psilocybin ↓ authoritarian views
Nisbet and Zelenski 2013	**Study 1 of 4:** *n* = 184, psychology undergraduate students, mean age 19.48 years (SD = 2.83), 67.4% female, Canada	Y	Y	N	N/A	↑ satisfaction with life ↑ self-acceptance ↑ purpose in life
	**Study 2 of 4:** *n* = 145 Canadian, middle managers, 24–70 years, 87 men, 56 women, 2 did not indicate sex, average age 42.37 (SD = 8.8), age range 24–70	Y	Y	N	N/A	↑ personal growth ↑ positive affect = autonomy
	**Study 3 of 4:** *n* = 354, majority (82.5%) first year university students, mean age 20.0 (SD = 4.36), 59.9% women	Y	Y	N	N/A	↑ well-being
	**Study 4 of 4:** *n* = 207 (*n* = 84 community participants and *n* = 123 student participants), 16–72 years, majority women, majority Caucasian	Y	Y	N	N/A	N/A
Nisbet, E.K.; Zelenski, J.M.; Murphy, S.A. 2009	**Study 1 of 2:** *n* = 831, Canadian undergraduate psychology students, mean age 19.84 (SD = 2.83), 124 females, 60 males *n* = 184 invited for follow-up session (test-retest)	Y	Y	N	N/A	N/A
	**Study 2 of 2:** *n* = 145, executives from government and private sector, average age 42.37 (SD = 8.8), 87 males, 56 females, 2 did not indicate gender, Canada	Y	Y	N	N/A	N/A
Puhakka et al., 2018	*n* = 914, mean age 17.8 years (SD = 0.5), men, City of Oulu in Northern Finland	N	N/A	N	N/A	↑ self-rated health ↓ alcohol intake ↓ smoking ↑ physical activity ↑ increased time in nature
Nature Relatedness Short Form (NR-6); Nisbet and Zelenski 2013 (*n* = 3 articles)						
Nisbet and Zelenski 2013	**Study 1 of 4:** *n* = 184, psychology undergraduate students, mean age 19.48 years (SD = 2.83), 67.4% female, Canada	Y	Y	N	N/A	↑ satisfaction with life ↑ self-acceptance ↑ purpose in life
	**Study 2 of 4:** *n* = 145 Canadian, middle managers, 24–70 years, 87 men, 56 women, 2 did not indicate sex, average age 42.37 (SD = 8.8), age range 24–70	Y	Y	N	N/A	↑ personal growth ↑ positive affect = autonomy
	**Study 3 of 4:** *n* = 354, majority (82.5%) first year university students, mean age 20.0 (SD = 4.36), 59.9% women	Y	Y	N	N/A	↑ well-being
	**Study 4 of 4:** *n* = 207 (*n* = 84 community participants and *n* = 123 student participants), 16–72 years, majority women, majority Caucasian	Y	Y	N	N/A	N/A
Richardson, Hussain, Griffiths 2018	*n* = 244, 90 males, 149 females, 5 did not disclose, primarily White (*n* = 199, 81.6%) U.K., U.S., Australia, Canada	N	N/A	N	N/A	↑ age and taking nature pictures ↑ selfie-taking ↑ time spent daily on smartphones
Zelenski and Nisbet 2014	*n* = 746, 331 students, 415 community, mostly female and Caucasian, New Zealand, U.S., Canada, U.K., Australia, and elsewhere	Y	Y	N	N/A	↑ happiness
Recalled Nature Connectedness (single item); Wyles et al., 2019 (*n* = 1 article)						
Wyles et al., 2019	*n* = 4515, 16 to 65+ years, 2359 female (52.2%), 2156 male (47.8%), England	N	N/A	N	N	↑ psychological benefits from urban greenspaces and coastal locations with designated status
Nature Connectedness; Michaelson et al., 2016 (*n* = 1 article)						
Piccininni et al., 2018	*n* = 20,697, 11–15 years, 9821 female, 10,942 male, Canada	N	N/A	N	N/A	↓ levels of psychological symptoms of females ↓ prevalence of psychomsomatic symptoms
Extended Inclusion of Nature in Self; Martin C. and S. Czellar 2016 (*n* = 1 article)						
Martin C. and S. Czellar 2016	**Study 2a of 4:** *n* = 107, average age = 21, 65% male, Switzerland	Y	Y	N	N/A	↑ green values ↑ materialistic value orientation ↑ environmental behavior ↓ aspiration index (value on achievement of extrinsic goals)
	**Study 2b of 4:** *n* = 585 participants, average age = 38, 42% male, Crowdflower.com users	Y	Y	N	N/A	↑ green values ↑ materialistic value orientation ↑ environmental behavior ↓ aspiration index (value on achievement of extrinsic goals)
	**Study 3a of 4:** *n* = 189, average age = 37, 45% male, Crowdflower.com users	Y	Y	N	N/A	= NEP ↑ NR-6 ↑ green values ↑ past green behavior = behavior (time) = behavior (ideas)
	**Study 3b of 4:** *n* = 178, average age = 35, 50% male, Crowdflower.com users	Y	Y	N	N/A	↑ love and care for nature (LCN) ↑ green values = NEP ↑ past green behavior = behavior (time) = behavior (ideas)
	**Study 4 of 4:** *n* = 232, average age = 23, Switzerland	N	N/A	N	N/A	N/A
Inclusion of Nature in Self (INS); Schultz 2002 (*n* = 8 articles)						
Beery 2013	*n* = 120, law students, Sweden	Y	Y	N	N/A	N/A
Colley, K. and T. Craig 2019	*n* = 236, average age 56.9 years (SD = 13.1), 64.4% from the city, 42.9% from urban fringe, 50% from small town, urban-rural transect following the river Dee, Northeast Scotland	Y	Y	N	N/A	N/A
Maurer and Bogner 2019	*n* = 464, Swiss German freshmen students, 66.5% female, average age 21.3 (SD = 3.1)	N	N/A	N	N	N/A
Nisbet and Zelenski 2011	**Study 1 of 2:** *n* = 150 Carleton University students, 16–48 years, 85 females, 56 male, 9 unspecified gender, Canada	N	N/A	N	N/A	↑ outdoor walks
	**Study 2 of 2:** *n* = 80	N	N/A	N	N/A	↑ outdoor walks
Nisbet and Zelenski 2013	**Study 3 of 4:** *n* = 354, majority (82.5%) first year university students, mean age 20.03 (SD = 4.36), 59.9% women (212 women, 142 men)	N	N/A	N	N/A	N/A
	**Study 4 of 4:** *n* = 207 (*n* = 84 community participants and *n* = 123 student participants), 16–72 years, majority women, majority Caucasian	N	N/A	N	N/A	N/A
Perkins, HE 2010	**Study 3 of 4:** *n* = 307, university business students, 18 years and older, 62% females, Australia	Y	Y	N	N/A	N/A
	**Study 4 of 4:** *n* = 261 tourists, 18–75 years, mean age 41 years, 42% male, 58% females, Australia	Y	Y	N	N/A	N/A
Richardson et al., 2016	*n* = 126, 111 females, 15 males, average age 43.2 (SD = 12.3), ages 22–71, U.K.	N	N/A	N	N/A	↑ health ↑ happiness ↑ connection to nature
Zelenski and Nisbet 2014	*n* = 746, 331 students, 415 community, mostly female and Caucasian, New Zealand, U.S., Canada, U.K., Australia, and elsewhere	Y	Y	N	N/A	N/A
Nature Inclusive Measure; St. John, MacDonald 2007 (*n* = 1 article)						
St. John, MacDonald 2007	*n* = 150 college students, mean age 31 years (SD = 10.35), ages 18–67) 118 female, 32 male, South Africa and Scotland	Y	Y	Y	Y	N/A

* Note: In the table above, *n* = number for the sample of the study, M = mean, SD = standard deviation, GOF statistics = goodness-of-fit statistics, Y = Yes, N = No, N/A = Not applicable, ↑ = positive association, ↓ = negative association, or equal sign = no change or equivalent outcome, no additional outcomes reported indicates there were no additional outcomes in addition to the psychometric outcomes reported.

## Appendix C

**Table A2:** ***** Summary of articles reviewing nature or environmental attitudes and values scales.

Last Name of Author(s), Year	General Demographics (n, Age, Gender, Location)	Reliability or Validity Reported	Acceptable Reliability or Validity	GOF Statistics Reported	Acceptable GOF Statistics	General Outcomes (↑, ↓, or =)
Attitudinal Commitment to Nature-based Activities; Allen and Meyer 1990 (*n* = 1 article)						
Asah et al., 2018	*n* = 236, 18+ years, 50.4% male, 42.4% female, 86.9% White, 20-state region of the Northeastern and Midwestern U.S.	Y	Y	N	N/A	N/A
Behavioral Commitment to Nature-based Activities; Asah et al., 2018 (*n* = 1 article)						
Asah et al., 2018	*n* = 236, 18+ years, 50.4% male, 42.4% female, 86.9% White, 20-state region of the Northeastern and Midwestern U.S.	Y	Y	N	N/A	N/A
Environmental Citizenship; Stern et al. 1999 (*n* = 1 article)						
Asah et al., 2018	*n* = 236, 18+ years, 50.4% male, 42.4% female, 86.9% White, 20-state region of the Northeastern and Midwestern U.S.	Y	Y	N	N/A	N/A
Biospheric Value (environmental); Schwartz 1992 (*n* = 1 article)						
Zibenberg et al., 2018	*n* = 583, university students (undergraduate and 21% graduate), average age = 20.5 years (SD 3.79), 73.4% female, Moscow, Russia	Y	Y	Y	Y	↑ private sphere behavior ↑ activism
Local Environmental Concern (pollution, hazards); Zibenberg et al., 2018 (*n* = 1 article)						
Zibenberg et al., 2018	*n* = 583 university students, 20.5 years (SD = 3.79), 73.4% female, Moscow, Russia	Y	Y	Y	Y	↑ local environmental concern ↑ activism
Environmental Attitudes; Guagnano and Markee 1995 (*n* = 1 article)						
Wright, Caserta, Lund 2003	*n* = 394, 70.5 years (SD = 8.1, range 55 to 99), 60% of the sample were men, Utah	Y	Y	N	N/A	↑ ascription of responsibility and awareness of consequences with environmental agentic disposition ↓ personal costs and environmental agentic disposition
The Population and Environment Scale; Harvey and Bell 1995 (*n* = 1 article)						
Wright, Caserta, Lund 2003	*n* = 394, 70.5 years (SD = 8.1, range 55 to 99), 60% of the sample were men, Utah	Y	Y	N	N/A	N/A
Children’s Ecological Behaviors Scale; Collado et al., 2015 (*n* = 1 articles)						
Collado et al., 2015	*n* = 107, 6–12 years, average = 9.35 years (SE = 1.52), 54.9% boys, urban camps in Spain	Y	Y	Y	Y	↑ emotional affinity towards nature ↑ ecological beliefs
Environmental Attitudes Inventory; Milfont and Duckitt 2010 (*n* = 1 article)						
AlMenhali et al., 2018	**Study 1 of 2:** *n* = 130, undergraduate students, 21–35 years, average 28 years (SD = 0.745), 92 females and 38 males, Abu Dhabi University	Y	Y	Y	Y	N/A
	**Study 2 of 2:** *n* = 130, 21–35 years old, average 25 years (SD = 0.785), 83 females and 47 males, Abu Dhabi University	Y	Y	Y	Y	N/A
Love and Care for Nature Scale; Perkins, HE 2010 (*n* = 1 article)						
Perkins, HE 2010	**Study 4 of 4:** *n* = 261 tourists, 18–75 years, average age 41 years, 42% male, 58% female), Gold Coast, Australia * more than 25% were international visitors	Y	Y	N	N/A	= openness to change values ↓ conservatism values
Environmental Behavior; Gkargkavouzi, A., S. Paraskevopoulos, and S. Matsiori 2018 (*n* = 1)						
Gkargkavouzi, A., S. Paraskevopoulos, and S. Matsiori 2018	*n* = 400, average age = 38.59 years (SD = 15.04), 51.9% female/48.1% male, 73.7% urban residents, Thessaloniki, Greece	Y	Y	N	N/A	↑ moderate-eco friends cluster (↑recycle several times per month, eco-friendly transportation choices, consume green way often, perform daily conservation activities in household) ↓ non-environmentalists (↓ involved in environmental policies, involved in civic actions, recycle, eco-friendly decisions)
Environmental Motives Scale (egoistic, altruistic, and biospheric concerns)						
Gkargkavouzi, A., G. Halkos, and S. Matsiori, A 2019	**Study 1 of 1:** *n* = 150, 87 females/63 males, mean age 40.32 (SD = 9.23), 22% college students, Greece	Y	Y	Y	Y	egoistic concerns ↓ CNS ↓ INS ↓ NEP altruistic concerns ↓ CNS ↓ INS ↓ NEP biospheric concerns ↑ CNS ↑ INS ↑ NEP
Gkargkavouzi 2019	**Study 2 of 2:** *n* = 400, Greek citizens, 38.36 years (SD = 14.29), 48% male/52% female, 73.7% urban residents, Greece	Y	Y	Y	Y	↓ egoistic concerns ↓ altruistic concerns ↑ biospheric concerns ↑ recycling behavior
Natural Environments and Feelings about Nature; Calogiuri 2016 (*n* = 1 article)						
Calogiuri 2016	*n* = 2168 adults, 18+ years, 50.4% male and 49.6% female, Norway	Y	Y	N	N/A	↑ physical activity
Perceived Importance of the Environment on Health and Well-being (Ropu Kaitiaki); Wiles et al., 2017 (109) (*n* = 1 article)						
Wiles et al., 2017	*n* = 671, (Maori (*n* = 267) born between 1920–1930, average 82.2 (SD = 2.6)) + (non-Maori (*n* = 404) born in 1925, average 84.6 (SD = 0.5)), the Bay of Plenty and Lakes District Health Boards (excluding Taupo regions) New Zealand	N	N/A	N	N/A	↑ well-being
Place Attachment; Jorgensen and Stedman 2001; Stedman 2003; Williams and Roggenbuck 1989; Williams and Vaske 2003 (*n* = 1 article)						
Colley, K. and T. Craig 2019	*n* = 236, average age 56.9 years (SD = 13.1), 51.8% female, 64.4% from the city, 42.9% from urban fringe, 50% from small town, urban-rural transect following the river Dee, North East Scotland	Y	Y	N	N/A	↑ walking distance ↑ wildness, management, and safety
Preferences for Nature Questionnaire; McMahan, E.A. and P. Josh 2017 (*n* = 1 article)						
McMahan, E.A. and P. Josh 2017	**Study 1 of 2:** *n* = 213, average age = 22.54 (SD = 7.83), 167 females, United States	Y	Y	N	N/A	preference of natural environments associated with: = socially desirable behaviors ↑ CNS = NEP-R
McMahan, E.A. and P. Josh 2017	**Study 2 of 2:** *n* = 168 adults, average age = 34.95 (SD = 11.43), 82 female/86 male, 79% Caucasian, Amazon’s Mechanical Turk workers, United States	Y	Y	Y	Y	preference of natural environments associated with ↑ socially desirable behaviors ↑ CNS ↑ NEP-R

* Note: In the table above, *n* = number for the sample of the study, M = mean, SD = standard deviation, GOF statistics = goodness-of-fit statistics, Y = Yes, N = No, N/A = Not applicable, ↑ = positive association, ↓ = negative association, or equal sign = no change or equivalent outcome, no additional outcomes reported indicates there were no additional outcomes in addition to the psychometric outcomes reported.

## Appendix D

**Table A3:** ***** Summary of articles reviewing cultural and spiritually based scales.

Last Name of Author(s), Year,	General Demographics (n, Age, Gender, Location)	Reliability or Validity Reported	Acceptable Reliability or Validity	GOF Statistics Reported	Acceptable GOF Statistics	General Outcomes (↑, ↓, or =)
Aboriginal Cultural Engagement Scale; Burgess et al., 2008; (*n* = 1 article)						
Berry et al., 2012	*n* = 27 total, 4 Aboriginal health workers (stage 1), *n* = 5 elders and Aboriginal consultants (stage 2), 13 Aboriginal consultants (stage 3), 5 expert Aboriginal consultants (stage 4), Australia	Y	Y	N	N/A	No additional outcomes reported
Awareness of Connectedness Scale; Mohatt et al., 2011 (*n* = 1 article)						
Mohatt et al., 2011	*n* = 284, 194 Alaska Native boarding school youth and 90 students from Yupʻik community, 12–18 years old, 164 female and 120 males, Southwest Alaska	Y	Y	Y	Y	↑ individual awareness
Cultural Connectedness Scale Snowshoe et al., 2015 (*n* = 2 articles)						
Crooks, et al., 2017	*n* = 105 survey, 53 male, 52 female; subset *n* = 28 interviewed First Nations Métis and Inuit youth, 11–14 years, Canada	Y	Y	N	N/A	↑ positive mental health and CCS with mentoring
Snowshoe et al., 2015	*n* = 319 First Nation, Métis and Inuit youth, 11–29 years (M = 15.3; SD = 2.3), 147 male, 162 female, 10 unspecified gender, Saskatchewan and Southwestern Ontario, Canada	Y	Y	Y	N	↑ wellbeing including traditions, spirituality, life satisfaction, sense of self (present and future), spiritual attendance
Hawaiian Cultural Scale; Hishinuma et al., 2000 (*n* = 1 article)						
Hishinuma et al., 2000	*n* = 3442 (2272 Hawaiian, 1170 non-Hawaiian), grades 9–12, Total: 49.4% male, 50.6% female, Native Hawaiians: 45.8% male, 54.2% female, Hawaiʻi	Y	Y	N	N/A	↑ valuing Hawaiian beliefs ↑ maintaining Hawaiian beliefs ↑ learning the Hawaiian way from family ↑ Hawaiian ancestry
Islamic Environmental Consciousness; Emari, Vazifehdoust, Nikoomaram 2017 (*n* = 1 article)						
Emari, Vazifehdoust, Nikoomaram 2017	*n* = 242 respondents, pollution industry participants, majority between the ages of 25–35 years, 54% female, 46% male, multiple Muslim countries	Y	Y	Y	Y	No additional outcomes reported
Multidimensional Model of Maori Identity and Cultural Engagement (MMM-ICE); Sibley and Houkamau 2013 (*n* = 1 article)						
Sibley and Houkamau 2013	*n* = 492 Māori, 14–75 years old (M = 30.61, SD = 14.40), 147 men, 331 women, 14 unreported gender, New Zealand	Y	Y	N	N/A	No additional outcomes reported
Pacific Identity and Wellbeing Scale- Revised (PIWBS-R); Manuela and Sibley 2015 (*n* = 1 article)						
Manuela and Sibley 2015	Subsample *n* = 521, Pacific peoples (Samoan, Tongan, Cook Island, Niuean, other Pacific), 18–74 years (M= 31.23, SD = 10.75), 387 female, New Zealand	Y	Y	Y	N	↑ speaking of Native language ↑ perceived familial wellbeing ↑ perceived societal wellbeing
Spiritual Attitude and Involvement List; deJager Meezenbroek et al., 2012 (*n* = 1 article)						
deJager Meezenbroek et al., 2012	Sample 1: *n* = 950 (students), 17–49 years old (M = 22), 14% male, Netherlands; Sample 2: *n* = 118 (healthy population), 39–83 years old (M = 54), 49% male, Netherlands; Sample 3: *n* = 348 (healthy interested), 25–85 years old (M = 42), 20% male, Netherlands; Sample 4: *n* = 153 (curative cancer), 25–84 years old (M = 57), 12% male, Netherlands	Y	Y	Y	Y	Connectedness with nature: ↑ connectedness with environment ↑ connectedness with others
Identification with Aboriginal Culture; Cameron 2004 (*n* = 1 article)						
Stroink and Nelson 2009	*n* = 20, 15–66 years of age (M = 32), 14 female, 6 male, First Nation communities of Ginoogaming and Aroland, Canada	N	N/A	N	N/A	↑ food knowledge ↑ food values ↑ life satisfaction ↑ social capital

* Note: In the table above, n = number for the sample of the study, M = mean, SD = standard deviation, GOF statistics = goodness-of-fit statistics, Y = Yes, N = No, N/A = Not applicable, ↑ = positive association, ↓ = negative association, or equal sign = no change or equivalent outcome, no additional outcomes reported indicates there were no additional outcomes in addition to the psychometric outcomes reported.

## Appendix E

**Table A4:** ***** Summary of articles reviewing paradigm-based scales.

Last Name of Author(s), Year	General Demographics (n, Age, Gender, Location)	Reliability or Validity Reported	Acceptable Reliability or Validity	GOF Statistics Reported	Acceptable GOF Statistics	General Outcomes (↑, ↓, or =)
New Ecological Consciousness; Ellis and Thompson 1997 (*n* = 1 article)						
Nisbet and Zelenski 2013	**Study 1 of 4:** *n* = 184, psychology undergraduate students, mean age 19.48 years, 67.4% female	Y	Y	N	N/A	N/A
New Ecological Paradigm for Children; Manoli, Johnson, and Dunlap 2008 (*n* = 1 article)						
Corraliza et al., 2013	*n* = 574, mean age = 11.32 years (SD = 1.39), 47.2% boys, Spain	Y	Y	Y	N	↑ pro-environmental behavior
Revised New Ecological Paradigm (NEP) Scale; Dunlap, Van Liere, Mertig, and Jones 2000 (*n* = 7 articles)						
Byrka et al., 2010	*n* = 468 students, 20–42 years (M = 23.19), 83.1% female, Germany	N	N/A	N	N/A	↑ use of natural environments for psychological restoration
Gkargkavouzi, Paraskevopoulos, and Matsiori 2018	*n* = 400, mean age = 38.59 years, 73.7% urban residents, Greece	Y	Y	N	N/A	↑ moderate-eco friends cluster (↑ recycle several times per month, eco-friendly transportation choices, consume green way often, perform daily conservation activities in household) ↓ non-environmentalists (↓ involved in environmental policies, involved in civic actions, recycle, eco-friendly decisions)
MacMillan Uribe, Winham, and Wharton 2012	*n* = 115, mean age = 42 years (SD = 12), 80.4% females, 95.2% identified as white, Arizona	N	N/A	N	N/A	= family involvement in food preparation ↑ sustainability behaviors
McMahan, E.A. and P. Josh 2017	**Study 2 of 2:** *n* = 168 adults, mean age = 34.95 years (SD = 11.43), 82 female, U.S.	Y	Mixed results	N	N/A	N/A
Nisbet and Zelenski 2013	**Study 1:** *n* = 184, psychology undergraduate students, mean age 19.48 years, 67.4% female	Y	Y	N	N/A	N/A
Perkins, HE 2010	**Study 1 of 4:** *n* = 261 tourists, 18–75 years old (average age 41 years), 58% females, Australia	Y	Y	N	N/A	N/A
vanRiper et al., 2019	*n* = 209, mean age = 45 years, 59.8% men, Australia	Y	Y	Y	Y	↑ motivations to engage with nature (achievement, similar people, enjoying nature, learning, escape)
New Environmental Paradigm Scale; Dunlap 2008; Dunlap, Van Liere, Mertig, and Jones 2000 (*n* = 3 articles)						
Gkargkavouzi 2019	**Study 1 of 2:** *n* = 150, Greek citizens, 87 females, mean age 40.32 (SD = 9.23), Greece	Y	Y	Y	Y	↓ egoistic concerns ↓ altruistic concerns ↑ biospheric concerns ↑ CNS (connectedness to nature)
	**Study 2 of 2:** *n* = 400, Greek citizens, 38.6 years (SD = 14.29), 52% female, Greece	Y	Y	Y	Y	↓ egoistic concerns ↓ altruistic concerns ↑ biospheric concerns ↑ recycling behavior
Mayer and Frantz 2004	**Study 1 of 4:** *n* = 60, mean age 31 years, 31 male, 29 female, U.S.	N	N/A	N	N/A	N/A
	**Study 2 of 4:** *n* = 102, psychology students, 60 females, U.S.	Y	Y	N	N/A	N/A
Reyes 2015	*n* = 46,234, Great Britain, U.S., Norway, Czech Republic, Slovenia, Bulgaria, Russia, New Zealand, Canada, Philippines, Japan, Spain	Y	Y	N	N/A	↑ environmental action

## Appendix F

**Table A5:** Summary of readability according to the Flesch Reading Ease and Flesch-Kincaid Grade Level.

Nature Connectedness, Attitudes, and Paradigm-Based Scales	Flesch Reading Ease	Flesch-Kincaid Grade Level
Behavioral Commitment to Nature-based Activities	42.1	10.5
Children’s Ecological Behaviors Scale	59.8	6.2
Connectedness to Nature Scale	70	7
Environmental Attitudes	48.7	8.4
Inclusion of Nature in Self (INS)	40.1	10.1
Nature Relatedness Scale	67.7	6.6
Nature Relatedness Short Form (NR-6)	67	7.1
New Ecological Consciousness	45.2	10.3
New Ecological Paradigm for Children	75.5	5.3
New Ecological Paradigm scale	58.4	8.2
Perceived Importance of the Environment on Health and Well-being (Ropu Kaitiaki)	77.1	4.5
Preferences for Nature Questionnaire	58.2	9.3
Recalled Nature Connectedness	N/A	N/A
The Population and Environment Scale	52.8	8.0
Two Major Environmental Values (2-MEV) Scale	66.7	6.7
Two Major Environmental Values (2-MEV) Scale	80.6	5.3
**Cultural and Spiritually Based Scales**	**Flesch Reading Ease**	**Flesch-Kincaid Grade Level**
Aboriginal Cultural Engagement Scale	35.6	11.1
Awareness of Connectedness Scale	61	6.9
Cultural Connectedness Scale	45.9	10.4
Hawaiian Cultural Scale	57.2	7.4
Multidimensional Model of Maori Identity and Cultural Engagement (MMM-ICE)	56.7	7.8
Pacific Identity and Wellbeing Scale-Revised (PIWBS-R)	58.3	7.3
Spiritual Attitude and Involvement List	76.1	5.3
